# Intralymphatic Administration of Adipose Mesenchymal Stem Cells Reduces the Severity of Collagen-Induced Experimental Arthritis

**DOI:** 10.3389/fimmu.2017.00462

**Published:** 2017-04-21

**Authors:** Pablo Mancheño-Corvo, Mercedes Lopez-Santalla, Ramon Menta, Olga DelaRosa, Francisca Mulero, Borja del Rio, Cristina Ramirez, Dirk Büscher, Juan A. Bueren, Juan Lopez-Belmonte, Wilfried Dalemans, Marina I. Garin, Eleuterio Lombardo

**Affiliations:** ^1^TiGenix SAU, Madrid, Spain; ^2^Division of Hematopoietic Innovative Therapies, Centro de Investigaciones Energéticas, Medioambientales y Tecnológicas (CIEMAT), Centro de Investigación Biomédica en Red de Enfermedades Raras (CIBER-ER), Madrid, Spain; ^3^Advanced Therapies Unit, Centro de Investigaciones Energéticas, Medioambientales y Tecnológicas (CIEMAT), Instituto de Investigación Sanitaria Fundación Jiménez Díaz (IIS-FJD), Madrid, Spain; ^4^Centro Nacional de Investigaciones Oncológicas (CNIO), Madrid, Spain; ^5^Grifols, Barcelona, Spain; ^6^Farma-Cros, Albacete, Spain; ^7^TiGenix NV, Leuven, Belgium

**Keywords:** adipose mesenchymal stem cells, intralymphatic route, collagen-induced arthritis, efficacy, immunomodulation

## Abstract

Mesenchymal stem cells (MSCs) are multipotent stromal cells with immunomodulatory properties. They have emerged as a very promising treatment for autoimmunity and inflammatory diseases such as rheumatoid arthritis. Previous studies have demonstrated that MSCs, administered systemically, migrate to lymphoid tissues associated with the inflammatory site where functional MSC-induced immune cells with a regulatory phenotype were increased mediating the immunomodulatory effects of MSCs. These results suggest that homing of MSCs to the lymphatic system plays an important role in the mechanism of action of MSCs *in vivo*. Thus, we hypothesized that direct intralymphatic (IL) (also referred as intranodal) administration of MSCs could be an alternative and effective route of administration for MSC-based therapy. Here, we report the feasibility and efficacy of the IL administration of human expanded adipose mesenchymal stem cells (eASCs) in a mouse model of collagen-induced arthritis (CIA). IL administration of eASCs attenuated the severity and progression of arthritis, reduced bone destruction and increased the levels of regulatory T cells (CD25^+^Foxp3^+^CD4^+^ cells) and Tr1 cells (IL10^+^CD4^+^), in spleen and draining lymph nodes. Taken together, these results indicate that IL administration of eASCs is very effective in modulating established CIA and may represent an alternative treatment modality for cell therapy with eASCs.

## Introduction

Mesenchymal stem cells (MSCs), which can be obtained from several adult tissues (1–5), have a potential use for cell therapy in inflammatory diseases based on their immunomodulatory properties and paracrine anti-fibrotic, anti-apoptotic, and pro-angiogenic effects (6–8). MSCs can modulate the function of a broad number of immune cells (9–11) by using multiple mechanisms including direct cell-to-cell contact and by means of soluble factors (9).

The therapeutic effects of MSCs have been demonstrated in experimental animal models of a wide variety of inflammatory diseases (10–17). The positive outcome of these preclinical studies supported the use of MSCs in clinical trials in different inflammatory diseases such as rheumatoid arthritis, systemic lupus erythematosus, and Crohn’s disease (16, 18–21).

The route of administration may impact the therapeutic effect of the treatment as the biodistribution and targeting of the cells to the inflammatory sites may vary depending on the route used (e.g., MSCs are mainly retained in the lungs when administered intravenously) (8, 22–24). In experimental animal models, mainly the intravenous (IV) or intraperitoneal routes of administration have been used (10, 25, 26), while the IV route of administration is routinely used in the clinical practice, allowing a systemic delivery of the therapeutic product. The intraperitoneal route is mainly restricted to preclinical studies. Interestingly, previous studies have demonstrated that systemically administered MSCs homed to lymphoid tissues associated with the inflammatory site where functional MSC-induced immune cells with regulatory phenotype (i.e., regulatory T cells) were increased, mediating the immunomodulatory effects of MSCs (10, 11, 27, 28).

Thus, we hypothesized that homing of MSCs to lymphoid tissues plays an important role in their immunomodulatory properties *in vivo* and that direct intralymphatic (IL) administration of MSCs could be an alternative and effective route for MSC therapies. Moreover, IL administration of MSCs could enhance their therapeutic effect since the number of MSCs that could be delivered inside the lymph node after intranodal infusion could be significantly greater than the number of cells that would reach the lymph nodes following their systemic delivery. In addition, this could also result in the need of lower doses of MSCs to achieve the same therapeutic effect, thus improving potentially the safety while reducing the production costs of the MSC-based therapies.

The IL route of administration has been reported to be safe and effective in human immunotherapy with dendritic cells or antigens improving the immune response against tumors and allergies, allowing a reduction in the number of administrations and doses (29–33). Importantly, the IL injection was well tolerated, practically painless, and enhanced patient convenience and compliance (34).

Here, we report the feasibility and efficacy of the IL administration of human expanded adipose mesenchymal stem cells (eASCs) in a mouse model of collagen-induced arthritis (CIA).

## Materials and Methods

### Generation and Characterization of Human Adipose-Derived MSCs

Human samples were obtained after informed consent as approved by the Spanish Ethics Committee of reference for the site of tissue procurement (Clínica de la Luz Hospital, Madrid, Spain). Human adipose tissue aspirates from healthy donors were washed twice with phosphate-buffered saline and digested with 0.075% collagenase (Type I, Invitrogen, Carlsbad, CA, USA). The digested sample was washed with 10% fetal bovine serum (FBS), treated with 160 mM NH_4_Cl to eliminate remaining erythrocytes and suspended in culture medium (Dulbecco’s Modified Eagle Medium, with 10% FBS). Cells were seeded in tissue culture flasks and expanded (37°C, 5% CO_2_) with change of culture medium every 3–4 days. Cells were transferred to a new flask when they reached 90% confluence. Cells were expanded up to duplication 12–14 and frozen. Experiments were performed with a pool of cells from three male and three female adult donors at population doublings 12–14. eASCs were thawed, seeded in tissue culture flasks, and trypsinized before administration to mice.

eASCs were defined according to the criteria of the International Society for Cellular Therapy (35), being positive for CD73 (AD2) and CD90 (5E10) from Becton Dickinson (Franklin Lakes, NJ, USA) and CD105 (43A3) from Biolegend (San Diego, CA, USA) and negative for CD14 (RM052) from Immunotech (Monrovia, CA, USA), CD19 (4G7), HLA-DR (L243), and CD34 (8G12) from Becton Dickinson and CD45 (J33) from Beckman Coulter (Brea, CA, USA) (Figure S1A in Supplementary Material).

For immunosuppression assays, peripheral blood mononuclear cells (PBMCs) were isolated by density centrifugation gradient using Ficoll-Paque Plus (GE Healthcare Biosciences AB, Uppsala, Sweden) from buffy coats provided by the National Transfusion Centre of the Comunidad Autonoma of Madrid, and splenocytes were obtained from C57/BL6 male mice.

For carboxyfluorescein diacetate N-succinimidyl ester (CFSE) labeling, PBMCs or splenocytes were washed extensively to remove FBS, resuspended in a 10 µM CFSE (Sigma-Aldrich, St Louis, MO, USA) solution (10^7^ PBMC or splenocytes per 200 µl of solution), and incubated under constant shaking at 37°C for 10 min. Reaction was stopped by adding ice-cold medium (RPMI + 10% FBS), and cells were washed three times with ice-cold phosphate buffer saline. Cells were then cultured overnight, and one aliquot was used to set up and control the FL-1 voltage for CFSE. After resting overnight, CFSE-labeled PBMCs were activated with the Pan T Cell Activation Kit (microbeads coated with anti-CD3, anti-CD2, and anti-CD28; Miltenyi Biotec, Auburn, CA, USA) following the manufacturer’s instructions. CFSE-labeled splenocytes were activated with anti-CD3 (Becton Dickinson) and IL-2 (Novartis, Basel, Switzerland). PBMCs or splenocytes (10^6^ cells/well) were cultured in 24-well plates alone or with eASCs (4 × 10^4^ cells/well; ratio 1:25 of eASC:PBMC or eASC:splenocytes) in a total volume of 2 ml of RPMI + 10% FBS. The 1:25 ratio was chosen because it provided a high inhibitory effect, on the basis of previous studies (36, 37). After 5 days for PBMCs and 3 days for splenocytes, cells were harvested, labeled with 7-AAD and anti-CD3 antibody and cell proliferation of the CD3^+^/7-AAD^−^ population (viable CD3 T lymphocytes) was determined by flow cytometry, according to loss of CFSE signal. Data were analyzed with the use of FCSExpress 4 (De Novo Software, Glendale, CA, USA) and BD CellQuest™ Pro analysis (Becton Dickinson) softwares. CaliBRITE beads (BD Bioscience, Erembodegem-Aalst, Belgium) were used to calibrate the acquisition events in the cytometer. As shown in Figure S1B in Supplementary Material, human eASCs strongly inhibited the proliferation of both human T lymphocytes and mouse splenocytes, demonstrating their immunomodulatory properties both in allogeneic and xenogeneic conditions.

### Induction and Evaluation of CIA

DBA/1 male mice, 8-week-old, were obtained from Janvier SAS, France.

Each mouse was injected subcutaneously in the tail with a first dose of an emulsion of Chicken Collagen Type II (1 mg/ml final concentration, Col II, Chondrex, Redmond, WA, USA) in Complete Freund’s Adjuvant (*Mycobacterium tuberculosis*) in a volume of 0.1 ml/animal. Twenty-one days after the first immunization with collagen II, a second injection was administered to each animal, again subcutaneously in the tail. In this occasion, the collagen suspension was made using Incomplete Freund’s Adjuvant (no *M. tuberculosis*). Clinical signs of arthritis were evaluated daily after the second immunization to determine clinical evidence of arthritis of the limb joints by macroscopic examination. The Arthritis Index Score was conducted until the end of the study. The severity of the arthritis was scored in both, front and hind, paws according to the following arthritis index scoring system: 0, no signs of arthritis; 1, swelling and/or reddening of the paw or 1 digit; 2, two groups of joints inflamed, swelling and/or reddening; 3, more than two groups of swelling and/or reddening joints; and 4, inflammation of the whole paw (38).

Paw edema was assessed daily as the volume of both, hind and front, paws by the use of a plethysmometer.

### IL Administration of eASCs in CIA Mice

In mice, the most suitable lymph nodes for injection are the inguinal ones due to their relatively easy access. Mice were anesthetized and, after depilation and disinfection of the inguinal region with 70% ethanol, a 6–8 mm incision was made. Injections were made with a Hamilton 30 gauge syringe. Three doses of 8 µl containing 5 × 10^4^ eASCs resuspended in Ringer’s Lactate (Grifols, Barcelona, Spain) were injected in both the right and left inguinal lymph nodes (total dose 1 × 10^5^/mouse) at day 1, day 8, and day 15. As a control, three doses of 8 µl of Ringer’s Lactate were injected in both the right and left inguinal lymph nodes at day 1, day 8, and day 15. Three doses of 200 µl of 2 × 10^5^ eASCs in Ringer’s Lactate were intravenously injected by the tail vein at day 1, day 8, and day 15. Mice were treated with eASCs when arthritis score was 2–4 according to Szabo et al. (38).

### Flow Cytometry Analysis

At day 22, mice were culled and cells were isolated from spleen and draining lymph nodes (inguinal and popliteal). Spleen and lymph nodes from each mouse were processed independently and disaggregated mechanically using a gentleMACS Dissociator AP^®^ (Miltenyi Biotec, Bergisch Gladbach, Germany). Cell counts were determined by Nucleocounter (Chemometec, Copenhagen, Denmark). Cells were surface stained with antibodies directed against with anti-CD4-FITC (GK1.5), anti-CD25-PE (REA568), and anti-CD3-PECy5 (REA641) (Miltenyi Biotec Bergisch Gladbach, Germany) and stained intracellularly with anti-Foxp3 (FKJ-16a, eBioscience, San Diego, CA, USA) using the Foxp3/Transcription factor staining buffer set (eBioscience) according to the manufacturer’s instructions. Appropriate isotype matched controls were included for the surface and intracellular staining.

For intracellular analysis of IL-10 cytokine expression, cells were stimulated *in vitro* with 5 ng/ml phorbol myristate acetate (Sigma-Aldrich, USA) and 500 ng/ml ionomycin (Sigma-Aldrich) for 4 h. GolgiPlug (BD Pharmingen) was added after 1 h. Cells were fixed and stained according to manufacturer’s instructions (Cytokine BD kit, BD Pharmingen). For intracellular staining, anti-IL-10 antibody (JES5-16E3, Becton Dickinson, BD Pharmingen) was used. Cells were acquired in a BD FACS CantoTM (Becton Dickinson). At least 10,000 events were acquired. Data were analyzed by BD FACSDiva™ (Becton Dickinson) and Flow Jo (FlowJo) softwares.

### Determination of Anti-Chicken Collagen II Antibodies and Cytokines in Plasma

Plasma concentration of autoantibodies against chicken collagen II was determined by ELISA (MD Bioproducts, Zurich, Switzerland) following manufacturer’s instructions.

Plasma levels of IL-6, TNF-α, IL-17, and IL-23 were determined by Luminex© multiplex technology (Life Technologies, Madrid, Alcobendas) following manufacturer’s instructions (Affymetrix-eBioscience, Santa Clara, CA, USA).

For *in vitro* detection of IL-10, IL-4, TNF-α, IL-17, and IFN-γ, splenocytes and lymph node cells were seeded in 96-well U-plates in 10% FBS supplemented RPMI (5 × 10^6^ and 1 × 10^6^ cells/well, respectively) and stimulated with chicken collagen II (25 µg/ml), and then supernatants were collected after 72 h of culture and cytokines determined as indicated above.

### Three-Dimensional Micro-Computed Tomography (micro-CT)

At day 50 after treatment, mice were sacrificed and hind paws were removed and fixed in 10% neutral buffered formalin. Before micro-CT scanning, paws were washed with running water for 15 min. The imaging of three-dimensional micro-CT was performed with GE eXplore Locus micro-CT scanner (GE Healthcare, Spain). Data were acquired at 27 µm isotropic voxel size with 720 projections by 360° scan, integration time of 2,000 ms with three frames, photon energy of 80 KeV, and current of 450 µA. The duration of imaging time was 100 min per scan and followed by 1 h of projection correction and volume reconstruction of three-dimensional representation. Three-dimensional render images of hind paws were generated through original volumetric reconstructed images by MicroView software (GE Healthcare, Spain). Comparable regions of interest of four metatarsal joints per hind paw (right and left, total eight joints per mouse) were determined. Bone mineral density (BMD = BM/BV mg/cm^3^) and trabecular mineral density (TMD) were quantified from micro-CT scans using GE MicroView software v2.2. Data are expressed as the BMD and TMD variation in untreated and eASC-treated CIA mice compared to healthy animals.

### Statistical Analysis

Data are expressed as mean plus the SEM. The mean data were analyzed with unpaired *U*-test (Mann–Whitney test) in the comparison between two groups or one-way analysis of the variance (Kruskal–Wallis test) with Dunn’s multiple comparison post-test where more than two groups were compared. A value of *p* < 0.05 was regarded as statistically significant. Analysis was performed using Graphpad software (Graphpad, La Jolla, CA, USA).

## Results

### Intralymphatic Administration of eASCs Effectively Reduces the Severity of Arthritis in CIA Mice

We and others have previously demonstrated the immunomodulatory capacity of human eASCs administered intravenously in experimental CIA mice (10, 11, 39, 40). In the present study, we investigated the feasibility and efficacy of the eASC administration into the lymph nodes in CIA mice and compared it with the IV administration.

**Figure 1:**
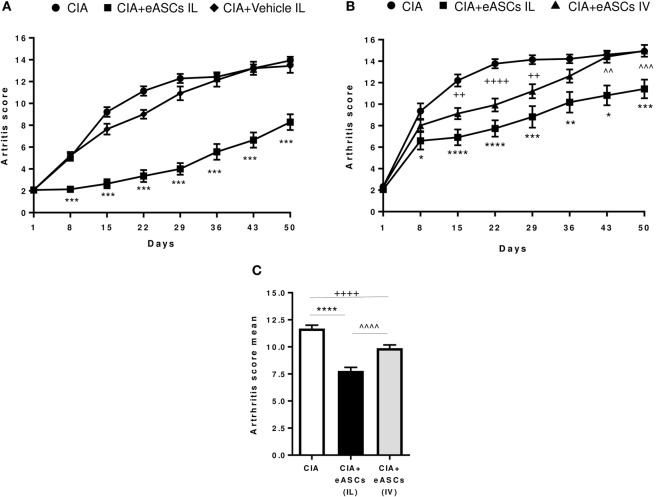
**Arthritis status of mice measured by arthritis score and arthritis score mean following intralymphatic (IL) and intravenous (IV) administration of eASCs**. **(A)** Collagen-induced arthritis (CIA) mice were either untreated or treated with three weekly infusions of Ringer’s lactate or Ringer’s lactate containing 5 × 10^4^ eASCs in the right and left inguinal lymph nodes (total dose: 1 × 10^5^) when mice showed signs of arthritis (arthritis score 2–4) (*n* = 14). **(B)** CIA mice were either untreated (*n* = 34) or treated with three weekly infusions of 5 × 10^4^ eASCs in the right and left inguinal lymph nodes (total dose: 1 × 10^5^, *n* = 34) or three weekly infusions of 2 × 10^5^ eASCs in the tail vein (*n* = 40) at day 1, day 8, and day 15 when mice showed signs of arthritis (arthritis score 2–4). The arthritis score (based on the number of swollen paws) was monitored for 7 weeks after treatment. **(C)** Mean of arthritis score and SEM after 7 weeks of follow-up in untreated and treated CIA mice (IL and IV). Results represent the mean and the SEM of all mice per time point. Significance was analyzed by Kruskal–Wallis and Mann–Whitney *U* tests. *p < 0.05, **p < 0.01, ***p < 0.001, and ****p < 0.0001, IL-treated CIA mice vs. CIA mice; ^++^*p* < 0.01 and ^++++^*p* < 0.0001, IV-treated CIA mice vs. CIA mice; and ^^^^*p* < 0.01, ^^^^^*p* < 0.001, and ^^^^^^*p* < 0.0001, IL-treated CIA mice vs. IV-treated CIA mice.

To first demonstrate the feasibility and efficacy of the IL administration of eASCs, CIA mice were injected at day 1, day 8, and day 15 in the right and left inguinal lymph nodes with either eASCs (5 × 10^4^ cells per lymph node; total dose per time point: 1 × 10^5^) or Ringer’s lactate (the vehicle of the eASCs), and the clinical score was monitored for 50 days after the treatment. In order to better reproduce the clinical situation, treatment was initiated when arthritis was already established (arthritis score 2–4) (day 1). As shown in Figure 1A, IL administration of eASCs significantly attenuated the severity of arthritis from day 8 to day 50. In contrast, the progression of the arthritis in CIA mice treated with vehicle was similar to untreated CIA mice. These results demonstrated that IL administration of eASCs, but not vehicle, had immunomodulatory effects and reduced the progression of arthritis in CIA mice.

We next investigated the efficacy of eASCs administered through the IL route compared to the IV route in CIA mice. In these studies, we used the lowest eASC dose per route of administration that, in our hands, efficiently modulated the progression of arthritis (minimal therapeutic dose per route, in our experimental settings). Thus, CIA mice were either untreated or treated at day 1, day 8, and day 15 with 2 × 10^5^ eASCs by the IV route or with 1 × 10^5^ eASCs by the IL route. As shown in Figure 1B, IV administration of eASCs significantly attenuated the severity of CIA from day 15 to day 29. Interestingly, the immunomodulatory effect of the IL administration showed a prolonged therapeutic effect that lasted for up to 50 days. The mean arthritis score for the whole study was 11.7 ± 0.3 for untreated, 7.8 ± 0.3 for IL eASC-treated, and 9.9 ± 0.3 for IV eASC-treated CIA mice, showing a higher efficacy of IL route compared to IV administration (Figure 1C).

Taken together, these results demonstrate that the IL administration of eASCs is feasible and could modulate the progression of arthritis in CIA mice potentially more efficiently than the IV administration.

### eASC Administration into the Lymph Nodes Reduced Bone Destruction in Arthritic Mice

**Figure 2:**
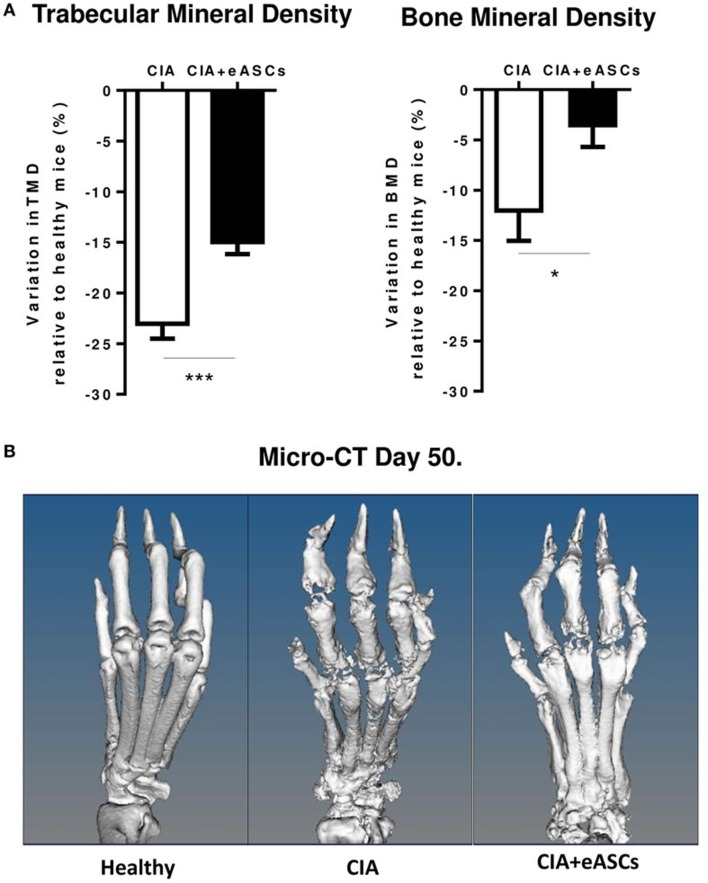
**Arthritis status of mice measured by trabecular mineral density (TMD) and bone mineral density (BMD), following intralymphatic (IL) administration of eASCs**. **(A)** Variation of TMD and BMD in untreated collagen-induced arthritis (CIA) mice and IL eASC-treated CIA mice relative to healthy mice 7 weeks after the treatment. Three representative mice per group were sacrificed at day 50, and TMD and BMD were determined by micro-computed tomography (micro-CT) in comparable regions of interest of four metatarsal joints per right and left hind paws (*n* = 8 joints per mouse, total *n* = 24 joints). **(B)** Micro-CT representative images of metatarsal joints of healthy, CIA, and IL eASC-treated CIA mice at day 50. Images show high-resolution three-dimensional rendering of micro-CT scans. Healthy: no sign of cortical bone destruction, CIA: high cortical bone destruction and loss of bone density in all phalanx and joints (presence of holes). CIA + eASCs: shows a more preserved structure of paws with less bone loss. Results represent the mean and the SEM of all joints. Significance was analyzed by Kruskal–Wallis and Mann–Whitney *U* tests. *p < 0.05 and ***p < 0.001.

In order to further confirm the therapeutic effect of the IL treatment with eASCs, joint damage on the hind paws of untreated and eASC-treated CIA mice was measured by means of micro-CT at day 50. As shown in Figure 2, the loss of TMD and BMD was significantly lower in eASC-treated CIA mice than in untreated CIA mice. The micro-CT images indicated that the administration of eASCs into the lymph nodes in CIA mice significantly reduced clinical symptoms, joint pathology, and local bone destruction in CIA mice.

**Figure 3:**
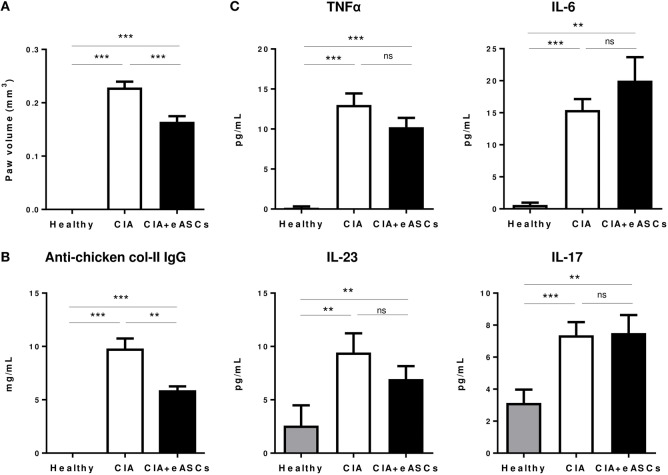
**Peripheral blood levels of anti-chicken collagen II antibodies and inflammatory cytokines**. Healthy, collagen-induced arthritis (CIA), and intralymphatic (IL) eASC-treated CIA mice were sacrificed at day 22 and hind paw edema **(A)**, peripheral blood levels of anti-chicken collagen II IgG **(B)**, and inflammatory cytokines TNFα, IL-6, IL-23, and IL-17 were measured by ELISA **(C)**. Data represent the mean and SEM (*n* = 14 mice). Significance was analyzed by Mann–Whitney test. **p < 0.01, ***p < 0.001, and ns, no significant.

We next determined the effect of eASCs on the generation of autoantibodies and cytokine secretion profile in peripheral blood. Mice were sacrificed 1 week after last infusion of eASCs (day 22), when the highest reduction in the arthritis score (Figure 1) and paw swelling (Figure 3A) was observed. As expected, anti-chicken collagen II antibodies, TNF-α, IL-6, IL-23, and IL-17 levels were increased significantly in CIA mice with respect to healthy mice (Figure 3). While IL eASC treatment reduced the level of anti-chicken collagen II IgG compared to untreated CIA mice, no significant effects were observed in the levels of circulating cytokines. This was similar to previous reports (41). However, a tendency to reduce the levels of TNF-α and IL-23 and some increment in IL-6 levels were observed in the eASC-treated CIA group.

These results suggest that the systemic inflammation in arthritic mice is not clearly affected upon IL treatment with eASCs and that their beneficial effects are mainly due to their effect in the lymphoid tissues and in the joints.

### Administration of eASCs into the Lymph Nodes Increases the Number of Immune T Cell Subsets with a Regulatory Phenotype in Spleen and Lymph Nodes

**Figure 4:**
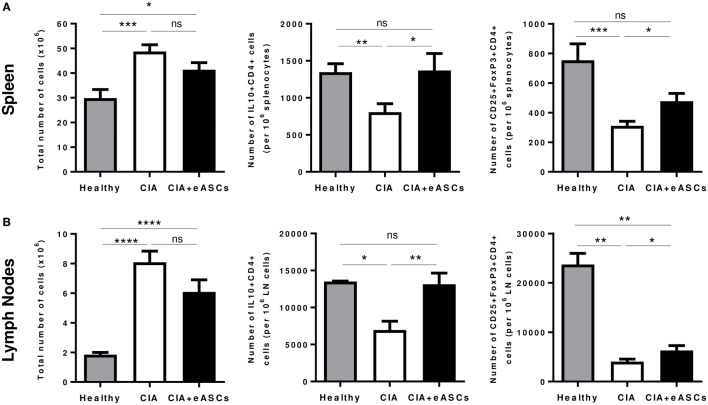
**Quantification of regulatory T cell populations identified as CD25^+^Foxp3^+^CD4^+^ and IL10^+^CD4^+^ in spleen and draining lymph nodes at day 22**. Intracellular expression of IL-10 cell suspensions was activated with phorbol myristate acetate and ionomycin for 4 h in the presence of GolgiPlug. After incubation, cells were harvested and stained on their surface with anti-CD4 and anti-CD25 monoclonal antibodies. For intracellular staining, cells were fixed, permeabilized, and stained with anti-IL10. The staining of Foxp3^+^CD4^+^ regulatory T cells was done using the Foxp3 transcription factor staining buffer set. Total number of cells, number of CD25^+^Foxp3^+^CD4^+^ and IL10^+^CD4^+^ T cells in spleen **(A)** and in lymph nodes **(B)** are represented by the mean and the SEM (*n* = 14). *p < 0.05, **p < 0.01, ***p < 0.001, ****p < 0.0001, and ns, no significant.

Several studies, in different experimental animal models of inflammation, have indicated that the therapeutic effect of eASCs is in part mediated by increasing immune T cells with a regulatory phenotype, such as regulatory T cells (CD25^+^Foxp3^+^ CD4^+^) and Tr1 cells (IL10^+^ CD4^+^) (10, 11, 42, 43). Therefore, we investigated the effects of the eASC administration into the lymph nodes on the levels of these regulatory T cell populations in spleen and lymph nodes (inguinal/popliteal) at day 22. A higher cellularity in the spleen and lymph nodes was found in CIA mice compared to healthy mice. In the eASC-treated CIA mice, total cell numbers tended to be reduced, although not significantly, with respect to untreated CIA mice (Figure 4).

As expected, CIA mice showed a significant reduction in IL10^+^CD4^+^ T cells and CD25^+^Foxp3^+^CD4^+^ T cells in spleen (Figure 4A; Figure S2A in Supplementary Material) and lymph nodes (Figure 4B; Figure S2B in Supplementary Material), compared to healthy mice. Interestingly, administration of eASCs into the lymph nodes increased the levels of IL10^+^CD4^+^ and CD25^+^Foxp3^+^CD4^+^ T cells in both tissues. Levels of IL10^+^CD4^+^ T cells in spleen and lymph nodes were similar to healthy mice suggesting that the eASCs tend to restore the homeostasis in the tissues.

Taken together, these results suggest that the inflammatory environment in CIA mice is accompanied by a broad reduction of regulatory T cells in lymphoid tissues and that the administration of eASCs in lymph nodes has a positive major impact on these two populations in spleen and draining lymph nodes.

### Administration of eASCs in Lymph Nodes in CIA Mice Decreases the Inflammatory/Regulatory Balance in Lymph Nodes

**Figure 5:**
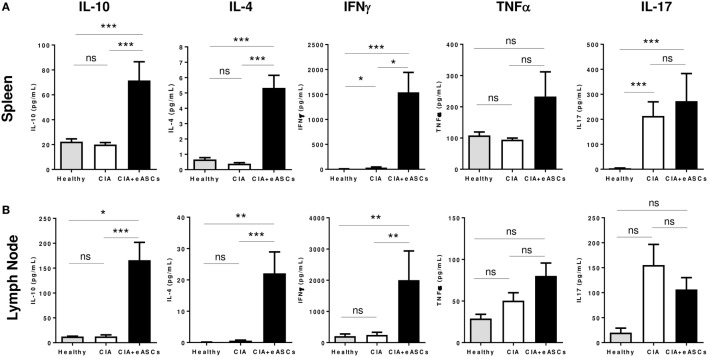
**Quantification of cytokine secretion in the supernatants of *in vitro* cultures of splenocytes and lymph node cells**. Splenocytes and lymph node cells harvested at day 22 were kept in culture for 72 h and stimulated with chicken collagen II, and then supernatants were collected. Levels of IL-10, IL-4, TNFα, IL-17, and IFNγ were determined by Luminex from spleen **(A)** and lymph node **(B)** cells. Results are represented by the mean and the SEM (*n* = 14). Significance was analyzed by Mann–Whitney test. *p < 0.05, **p < 0.01, ***p < 0.001, and ns, no significant.

The secretion of IL-10, IL-4, IFNγ, TNFα, and IL-17 cytokines in the supernatant of *in vitro* cultured splenocytes and draining lymph node cells in response to collagen II were determined (Figure 5). Increased levels of IL-10, IL-4, and IFNγ were found to be increased in eASC-treated CIA mice compared to CIA and healthy mice (Figure 5). Additionally, no clear effects were observed in the secretion of TNFα and IL-17 in both tissues.

**Figure 6:**
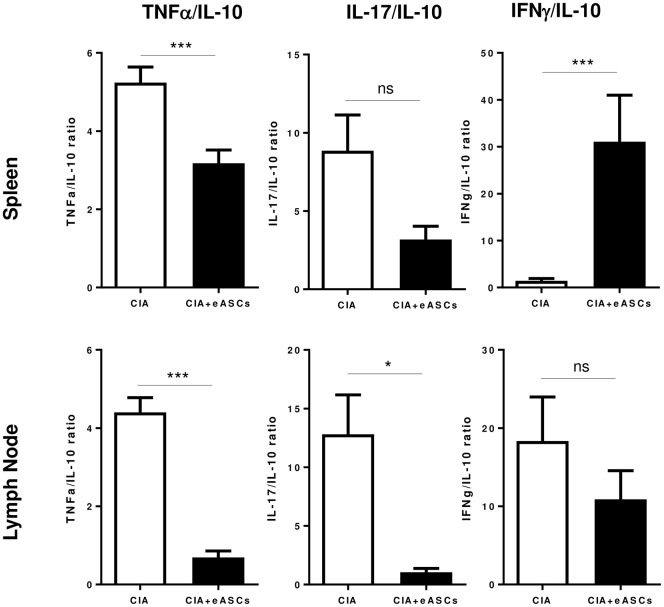
**The inflammatory/regulatory balance evaluated in spleen and lymph nodes**. Inflammatory/regulatory balance was calculated on the basis of TNFα/IL-10, IL-17/IL-10, and IFNγ/IL-10 ratios of secreted cytokines. Results are represented by the mean and the SEM. Significance was analyzed by Mann–Whitney test. *p < 0.05 and ***p < 0.001.

Interestingly, the inflammatory/regulatory balance in spleen and lymph nodes, as shown by the TNFα/IL-10 and IL17/IL-10 ratios, was reduced in eASC-treated CIA mice with respect to the untreated CIA mice (Figure 6). The inflammatory/regulatory balance measured by IFNγ/IL-10 ratio was significantly increased in the spleen in eASC-treated CIA mice with respect to untreated CIA mice. This was in contrast to the lymph nodes where IFNγ/IL-10 ratio was decreased in eASC-treated CIA mice with respect to untreated CIA mice. These results are in agreement with previous data obtained by us and others (10, 44).

These results further confirm that the administration of eASCs into the lymph nodes reduces the inflammatory/regulatory balance mainly in the draining lymph nodes of CIA mice where the eASCs were delivered.

## Discussion

Several studies have reported the detection of infused MSCs within the lymphatic system. MSCs administered systemically in inflamed mice can migrate to the draining lymph nodes and spleen where increased number of regulatory myeloid and lymphoid cells efficiently controls the progression of inflammation (28, 45). Additionally, the navigation of native MSCs, mobilized from adipose tissue, through the lymph fluid homing into active lymph nodes has been reported (27).

Based on these observations, we thus hypothesized that the homing of MSCs to lymphoid tissues represents a key step in their immunomodulatory effects *in vivo* and, therefore, injecting eASCs directly within the lymphatic system through the inguinal lymph node delivery may increase their availability within the lymphatic system and, hence, improve their efficacy *in vivo*. Here, we report the therapeutic efficacy of the IL administration of human eASCs in mice with established CIA. To our knowledge, this is the first time that modulation with MSCs of an ongoing inflammation by directly targeting immune responses within the lymph nodes has been demonstrated.

The highest eASC dose that can be injected in the inguinal lymph nodes of mice is approximately 1.6 × 10^5^ eASCs per lymph node (total dose of 3.2 × 10^5^ eASCs/mouse). This cell dose represents a compromise between the highest concentration of eASCs that can be used without affecting cell viability and the maximum volume of the cell suspension that can be injected in the inguinal lymph nodes without disrupting the functionality of the lymphoid tissue (approximately 8 µl) (33, 46).

Preliminary experiments were performed to identify the optimal dosing schedule and cell dose with the highest immunomodulatory effects. We found that 1 × 10^5^ eASCs/mouse had comparable therapeutic effects than 3.2 × 10^5^ eASCs/mouse, so all further studies were carried out with 1 × 10^5^ cells, which was considered to be the minimal therapeutic dose under these experimental conditions. Three administrations with a week interval were considered to be the best dosing schedule. The interval of 1 week was used since the IL injections in mice required a surgical procedure, and 1 week was the time period needed for complete healing of the incision.

Three IL administrations 1 week apart of 1 × 10^5^ eASCs, during the onset of the arthritis (arthritis score 2–4), significantly reduced the severity of the disease (clinical score and joint damage) until the end of the follow-up period of 50 days from the initiation of the treatment, showing a sustained therapeutic effect for up to 35 days from the last infusion of eASCs (Figure 1). This result suggests that the IL administration of eASCs is very effective in modulating, for a prolonged period of time, a systemic inflammatory response with a low dose eASCs. Remarkably, the direct injection of xenogeneic human eASCs in the lymphatic system of immunocompetent mice did not impair the therapeutic capacity of eASCs in CIA, confirming a positive tolerability profile of these cells. Nevertheless, the potential immunogenicity of MSCs is currently under evaluation as specific cellular and humoral immune responses against allogeneic MSCs have been reported in experimental animal models (47). How far these allo-immune responses may impair the MSC modulatory activity itself is not fully known. Overall, immunogenicity of allogeneic MSCs remains poorly understood and deserves further investigation and close monitoring in clinical trials.

Compiling data have demonstrated that mouse and human eASCs differ in some of their mechanisms of immunomodulation. Preclinical models of arthritis and colitis using human eASCs allowed dissection of pathways shared by the human and mouse systems (28, 48–50). Moreover, we have recently shown that eASCs are short-lived after *in vivo* administration (40), even when used in a syngeneic setting (8), indicating that eASCs, regardless the MHC context, can prime host immune cells thorough an array of not fully understood specific molecular mechanisms, which, in turn, adopt a regulatory phenotype.

Interestingly, after the IL administration of eASCs, a systemic therapeutic effect was observed. As expected, untreated CIA mice had high levels of anti-chicken collagen II antibodies and inflammatory cytokines in peripheral blood with respect to healthy mice (Figure 3). The IL administration of eASCs reduced significantly the levels of antigen-specific antibodies to chicken collagen II. In contrast, the levels of inflammatory cytokines were not considerably reduced. These results are in contrast to previous observations (42). This could be related to the difference in the time point measured (day 22 vs. day 12 after initiation of treatment) and the route used for administering the eASCs.

In addition, the cellularity of the spleen and lymph nodes, which was significantly increased in CIA mice with respect to healthy animals, was also reduced by eASC treatment, further confirming their anti-inflammatory effects. These effects were accompanied with increased levels of regulatory T cells, Tr1 cells and increased production of anti-inflammatory cytokines (i.e., IL-4 and IL-10). This is in agreement with what has been described previously using the intraperitoneal or the IV infusion of eASCs (10, 11, 51). Interestingly, treatment of CIA mice with regulatory T cells isolated from mice treated with human ASCs significantly attenuated arthritis, indicating that these cells have indeed a regulatory phenotype *in vivo* (42). We speculate that a similar mechanism may underlie the therapeutic benefit of eASCs administered intralymphatically. Dissecting the cellular and molecular mechanisms by which IL-administered ASCs reduce the severity of arthritis deserves further investigation.

Several administration routes such as IV, intraarticular, and intraperitoneal, in preclinical *in vivo* models, have been used for MSC administration (25, 40). In general, the IL route is currently being used in immunotherapy protocols of allergies and cancer as a mean of inducing stronger immunogenicity than other routes like subcutaneous or sublingual (29, 31). An interesting aspect of our study is the fact that the IL route can also be used for modulating ongoing immune responses systemically.

Biodistribution can be an important parameter that could have a major impact on the therapeutic effects of eASCs. It has been reported that MSCs, intravenously administered, accumulate almost exclusively in the lungs (22, 23). As described by Lopez-Santalla et al. (submitted manuscript), we have investigated the biodistribution and efficacy of eASCs administered intralymphatically in healthy and mice with TNBS-induced colitis using luciferase-expressing eASCs (Luci-eASCs) and found that there is a short eASCs persistence of around 7 days being mainly detected in the lymphatic system, mostly in the inguinal lymph nodes, with low levels of bioluminescence in the organs such as spleen, gut, liver, etc. Interestingly, when Luci-eASCs were infused intranodally in colitic mice, a significant increase in the bioluminescence signal was detected in the main organs, in particular in the inflamed gut, suggesting some trafficking of the Luci-eASCs toward the inflamed tissues (Lopez-Santalla et al. submitted). Notably, in Luci-eASC-treated colitic mice the severity of colitis was attenuated. These results further support the idea that the IL administration of eASCs can represent a realistic alternative route of administration for the treatment of inflammatory diseases, including rheumatoid arthritis and colitis.

In our study, a low dose of 1 × 10^5^ eASCs administered intralymphatically attenuated the progression of arthritis up to day 50. Previous studies using the intraperitoneal or IV route of administration required a higher dose (one or several doses of 1 × 10^6^ cells) to reduce the severity of arthritis (10, 11, 42). These results suggest that eASCs administered intralymphatically could have a better therapeutic effect. A significant delay in the progression of the CIA was observed using a cell dose of 2 × 10^5^ eASCs/mouse when cells were administered intravenously (the lowest cell dose able to effectively modulate established CIA in our experimental settings). These results suggest that the IL administration of eASCs may have better therapeutic effects than the IV route since a lower cells dose of 1 × 10^5^ eASCs/mouse was sufficient to modulate the progression of CIA for a longer period of time (up to 50 days). Further studies are needed in order to confirm these observations.

In our studies, IL treatment of arthritic mice with eASCs improved the trabecular bone parameter and mitigated bone loss. Based on the present data, it is reasonable to speculate that increased osteoclastogenesis in pathological conditions such as CIA can be delayed as a consequence of the treatment with eASCs. This is in agreement with previous studies conducted by Garimella and collaborators where the authors demonstrated that eASC treatment ameliorated inflammation-induced systemic bone losses in CIA mice by reducing osteoclast precursors and promoting immune tolerance (52).

In summary, our results show that the IL administration of eASCs attenuated the severity and progression of arthritis. Among the mechanisms underlying these effects were the induction of an adaptive regulatory T cell signature characterized by the increased numbers of IL10^+^CD4^+^ and CD25^+^Foxp3^+^CD4^+^ T cells in lymph nodes and spleen, which contributed to the development of an IL10-driven anti-inflammatory response during arthritis leading to a reduced inflammatory/regulatory balance in tissues. These observations are in agreement with previous studies in preclinical models of arthritis and in clinical trials using eASCs administered by other routes of administration suggesting that the mechanism underlying these observations may be similar (10, 20, 43, 53, 54).

Our results support the notion that the IL administration of eASCs can be a potential alternative route of administration for the treatment of inflammatory diseases. Future studies are warranted and should be oriented toward defining the full potential of IL infusion of eASCs, comparing the efficacy of the IL route with the IV route and the mechanisms underlying the modulation of immune responses.

## Conclusion

The IL administration of eASCs is effective for treating established experimental arthritis and may represent an efficient alternative route of administration for MSC-mediated therapy in inflammatory and autoimmune diseases.

## Ethics Statement

All experiments were performed in accordance with the corresponding regulations regarding experimental animal welfare (RD 223/1988 and Directive 2010/63/EU protocols) and approved by the Institutional Animal Care and Use Committee at the University of Albacete, Spain.

## Author Contributions

Conception and/or design of the work: PM-C, ML-S, OD, DB, BR, CR, JB, JL-B, WD, MG, and EL; acquisition of data for the work: PM-C, ML-S, RM, FM, BR, CR, JL-B, MG, and EL; analysis and interpretation of data for the work: PM-C, ML-S, RM, OD, FM, BR, CR, DB, JL-B, WD, MG, and EL; manuscript writing: PM-C, ML-S, MG, and EL; revision of manuscript for important intellectual content: RM, OD, FM, DB, JB, and WD; reviewed the manuscript and gave final approval for the work: all authors.

## Conflict of Interest Statement

PM-C, RM, OD, CR, BR, WD, and EL are full time employees of TiGenix. DB is full time employee of Grifols. JL-B is full time employee of Farma-Cros.

## Supplementary Material

Figure S1 **Characterization of eASCs**. **(A)** eASCs were characterized by flow cytometry using antibodies against CD14, CD19, CD34, CD45, HLA-DR, CD73, CD90, and CD105 (gray color). The corresponding isotype controls for each surface marker are shown (white color). Histograms shown are representative of three experiments. **(B)** Representative histograms of T-lymphocyte proliferation are shown. Carboxyfluorescein diacetate N-succinimidyl ester (CFSE)-labeled peripheral blood mononuclear cells (PBMCs, above) were activated with the Pan T Cell Activation Kit (microbeads coated with anti-CD3, anti-CD2, and anti-CD28) and CFSE-labeled splenocytes (below) with anti-CD3 and IL-2 in the absence or presence of human eASCs. PBMCs or splenocytes (10^6^ cells/well) were cultured in 24-well plates alone or with eASCs (4 × 10^4^ cells/well; ratio 1:25 of eASC:PBMC or eASC:splenocytes). Proliferation of the viable CD3^+^ T cells was monitored after 5 days for PBMCs and 3 days for splenocytes by flow cytometry.

Figure S2 **Quantification of IL10^+^CD4^+^ and Foxp3^+^CD4^+^ regulatory T cell populations in spleen and lymph nodes by flow cytometry at day 22 of collagen-induced arthritis (CIA) and intralymphatically eASC-treated CIA mice**. **(A)** Gating strategy for quantification of regulatory T cells subsets. The live cells were gated in the forward scatter/side scatter corresponding to the lymphocyte population. The intracellular expression of IL-10 and Foxp3 were determined separately within the CD3^+^CD4^+^ T cell population. Representative dot plots of the corresponding isotype controls are shown **(A)**. Representative dot plots of IL10^+^CD4^+^ T cells **(B)** and Foxp3^+^CD4^+^ regulatory T cells **(C)** in spleen and lymph nodes are shown.
