# Supplementary figures and images for: Intralymphatic Administration of Adipose Mesenchymal Stem Cells Reduces the Severity of Collagen-Induced Experimental Arthritis
Source: Front Immunol. 2017 Apr 21;8:462. doi: 10.3389/fimmu.2017.00462 (PMC5399019; doi:10.3389/fimmu.2017.00462)

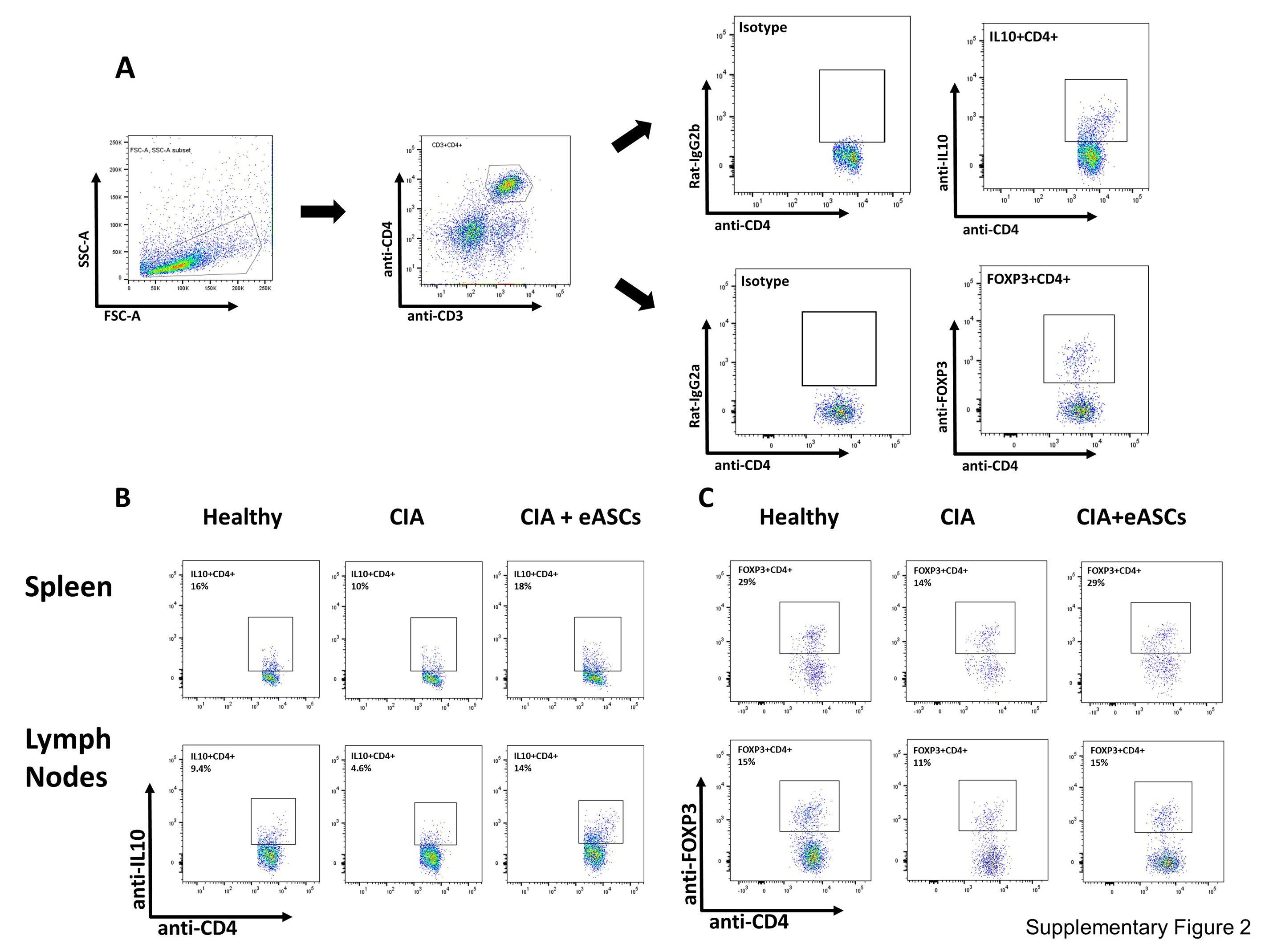

Supplement: Figure S2 — Quantification of IL10+CD4+ and Foxp3+CD4+ regulatory T cell populations in spleen and lymph nodes by flow cytometry at day 22 of collagen-induced arthritis (CIA) and intralymphatically eASC-treated CIA mice. (A) Gating strategy for quantification of regulatory T cells subsets. The live cells were gated in the forward scatter/side scatter corresponding to the lymphocyte population. The intracellular expression of IL-10 and Foxp3 were determined separately within the CD3+CD4+ T cell population. Representative dot plots of the corresponding isotype controls are shown (A). Representative dot plots of IL10+CD4+ T cells (B) and Foxp3+CD4+ regulatory T cells (C) in spleen and lymph nodes are shown. [file Image_2.TIF]
